# Evaluating the detectability of methane point sources from satellite observing systems using microscale modeling

**DOI:** 10.1038/s41598-022-20567-z

**Published:** 2022-10-19

**Authors:** Piyush Bhardwaj, Rajesh Kumar, Douglas A. Mitchell, Cynthia A. Randles, Nicole Downey, Doug Blewitt, Branko Kosovic

**Affiliations:** 1 National Center for Atmospheric Research, Boulder, CO USA; 2 ExxonMobil Upstream Research Company, Spring, TX USA; 3 ExxonMobil Research and Engineering Company, Annandale, NJ USA; 4 Earth System Sciences, LLC, Albuquerque, NM USA

**Keywords:** Climate change, Environmental impact, Carbon cycle

## Abstract

This study evaluates the efficacy of current satellite observing systems to detect methane point sources from typical oil and gas production (O&G) facilities using a novel very high-resolution methane concentration dataset generated using a microscale model. Transport and dispersion of typical methane emissions from seven well pads were simulated and the column enhancements for pseudo satellite pixel sizes of 3, 1, and 0.05 km were examined every second of the 2-h simulations (7200 realizations). The detectability of plumes increased with a pixel resolution, but two orders of magnitude change in emission rates at the surface results only in about 0.4%, 1.6%, and 47.8% enhancement in the pseudo-satellite retrieved methane column at 3, 1, and 0.05 km, respectively. Average methane emission rates estimated by employing the integrated mass enhancement (IME) method to column enhancements at 0.05 km showed an underestimation of the mean emissions by 0.2–6.4%. We show that IME derived satellite-based inversions of methane emissions work well for large persistent emission sources (e.g., super emitters), however, the method is ill-suited to resolve short-term emission fluctuations (< 20 min) in typical well site emissions due to the limitations in satellite detection limits, precision, overpass timing, and pixel resolution.

## Introduction

Methane is emitted from a variety of anthropogenic sources with the global fossil fuel industry (oil, gas, and coal) contributing about 15–22% of the total atmospheric methane budget^1^. Natural gas and petroleum systems contribute about 28% of the total US anthropogenic methane emissions^2,3^. Since methane is a potent greenhouse gas (global warming potential 28 times that of CO_2_ over a period of 100 years^4^), accurate quantification of atmospheric methane sources is essential to understanding and mitigating methane emission impacts on climate. Recently, many studies explored how satellites, with their advantages of global coverage at high spatiotemporal resolution, might help to detect very large methane leaks from anomalous oil and gas (O&G) operations, termed super-emitters^5–7^. However, less attention has been paid to the ability of satellites to detect routine O&G emissions associated with day-to-day operations. Here, we simulate the transport and dispersion of methane emitted from seven representative well pads at a very high spatial resolution (10 × 10 m^2^) using the Large Eddy Simulation capability of the Weather Research and Forecasting (WRF-LES) model. This WRF-LES output archived every second is used to derive synthetic methane columns at different spatial resolutions to (1) understand the detectability of the methane plumes as a function of satellite resolution, and (b) use the Integrated Mass Enhancement (IME) method^5^ to understand at what time scales the satellite measurement can resolve temporal fluctuations in methane emissions over an O&G field encompassing multiple sources. Methane retrievals and top-down emission estimates based on current polar-orbiting satellites are representative of the time of measurement. Future geostationary satellites are expected to address this limitation by continuously staring over a region of interest. Here, we aim to understand whether the IME method can detect short-term fluctuations if the future geostationary satellites can retrieve methane every second. To understand the impact of integration time and quantify the source rate errors, we also estimated methane emissions using 10-min averaged plumes. A 10-min integration time is selected because previous studies have shown that error in WRF-LES simulated wind speed becomes less than 1 m/s for averaging time of 10 min or higher.

## Methodology

### Model setup

We used the WRF-LES model version 3.8.1 to simulate methane plumes emitted from seven well pads^6^ located in a region representative of the Barnett Shale, Texas (33°N, 98.2°W). Briefly, we configured 2 nested domains covering the areas of 10.3 km × 12.5 km and 6.9 km × 8.3 km at 30 m and 10 m horizontal grid spacing, respectively. The model has 121 vertical levels up to 2 km with ~ 3 m vertical resolution near the surface that increases to ~ 27 m near 2 km. The model simulates daytime convective conditions and is initialized and forced at the southern boundary of the outer domain with steady southerly winds at 5 m/s (see Saide et al.^6^ for full details). Different real-world meteorological conditions (a combination of sensible heat flux and prevailing winds) could also impact the emissions estimation.

**Figure 1:**
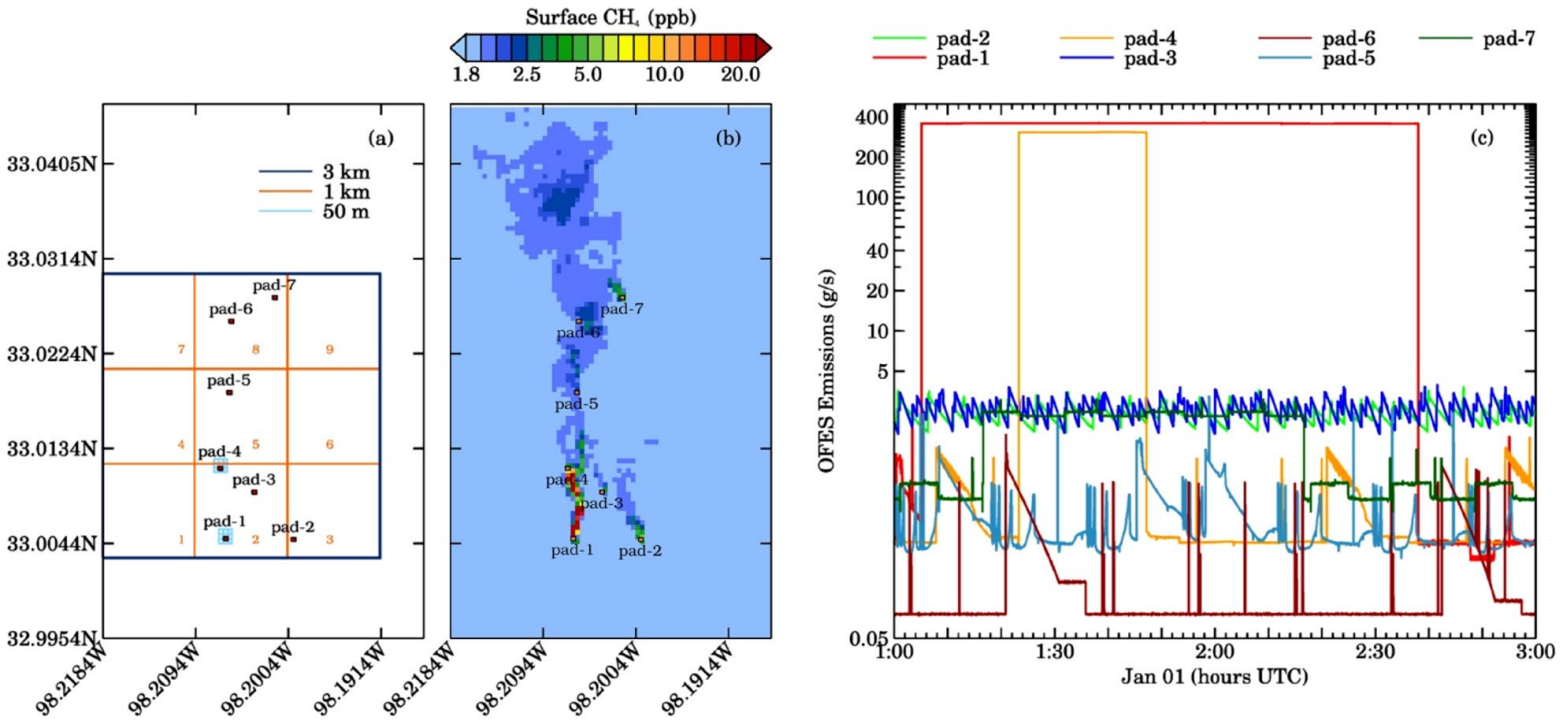
(**a**) High-resolution (10 m) domain of WRF-LES setup over the Barnett Shale in Texas. Three boxes represent pseudo satellite footprints at 3 km (blue), 1 km (orange) and 50 m (light blue around pads 1 and 4) spatial resolution. The location of seven oil pads is also indicated in the domain with each having multiple sources. (**b**) Spatial distribution of surface methane mixing ratio at 1:20:00 h UTC. (**c**) Input OFES emissions from seven well pads during the two-hour simulation period.

The simulations were run for three hours with the first hour discarded as spin-up. Methane concentrations are saved every second from the 10 m domain. The study region has seven hypothetical well pads with typical spacing for the Barnett shale (Fig. 1). We include 22 methane tracers to represent different emission sources (Table S1) located in the inner domain^6^. Time-varying methane emissions from these 22 sources were generated every second by the Earth System Science (ESS) oil field emission simulator (OFES)^8^. In addition, a tracer representing the background methane concentration, and two additional constant unit emission rate tracers were included to analyze the impact of turbulence.

### Calculation of methane columns

Total methane columns are calculated by integrating the WRF-LES simulated methane profiles extended to the top of the atmosphere (4.5 × 10^–6^ hPa) using the Whole Atmosphere Community Climate Model^9^ (WACCM) climatology for Jan 2018. Methane columns are calculated for three pseudo satellite pixel sizes, 3 × 3 km^2^, 1 × 1 km^2^, and 50 × 50 m^2^ by averaging over adjacent grid points (i.e., averaging over 5 × 5 pixels for the 50 m pixel size). These resolutions roughly equate to the conceptual (GeoFTS), proposed (MethaneSAT), or ongoing (GHGSat-D) satellite missions^10^. The percent enhancement in methane columns relative to background is then calculated for each pseudo pixel. The satellite retrieved methane column in short wave infrared region (SWIR) is based on measurements of solar backscatter radiation at 1.65 or 2.3 μm^10^. The retrieved columns depend on averaging kernel vector, which provides sensitivity of measurement signal at any particular altitude; for SWIR measurements the averaging kernel vector is very close to unity^10^. Satellite retrievals are typically noisy, therefore, we considered three different scenarios using 50 m resolution WRF-LES derived pseudo-measurements, first being noise free (“best case”) scenario, and with additional 1%, and 5% instrument noise, respectively. This variable instrument noise (1–5%) is randomly chosen (is assumed to be gaussian in nature) and it is added to each pseudo-satellite pixel to represent typical satellite column retrievals at different instrument precisions. Figure S1 shows spatial distributions of a methane columns with four different instrument noise levels from noise free to 5%. The plume detection is also sensitive to the instrument noise and a recent study using GHGSat-D suggested, that the methane column fields retrieved using the above method could have errors of 8–25% due to aerosol extinction^11^.

The input emissions include two liquid unloading events at well pads 1 and 4, which are characterized by methane emissions about two orders of magnitude greater (Fig. 1c) than emissions from other sources. To understand how such large sudden changes in methane emissions at the surface reflect in satellite methane column measurements, we consider four emission scenarios (Table S1). The first scenario considers the column enhancement resulting from all emission sources (ALL). The second scenario considers all sources except the liquid unloading emissions at pads 1 and 4 (NOPAD14). The third and fourth scenarios exclude the liquid unloading emissions from pad 1 (NOPAD1) and pad 4 (NOPAD4), respectively.

### Integrated mass enhancement (IME) and source rate

The behavior of methane plumes from O&G production wells depends on atmospheric turbulence, advection, and the strength of the emission source. Different methods exist to infer the source rates of such plumes, namely gaussian plume inversion^12^, source pixel method^10^, cross-sectional flux method^10,13,14^ and the IME method^5,10,14,15^. The nature of instantaneous plumes and their variability in downwind transport suggests the IME and cross sectional methods to be the most appropriate^5^ source rate estimation methods. Both the methods require determination of local/effective wind speed but the IME method showed a lower error in effective wind speed determination^5,16^.

Therefore, we used the IME method for source rate estimation in a region of 50 × 50 m^2^ resolution pseudo satellite pixels. The 50 m pixels were used because the larger pseudo pixel sizes had minimal enhancements above background making it difficult to discern emissions above typical satellite noise levels as will be shown later. The method is (also described in the supplementary text) based on Varon et al.^5^, and relates the source rate to the total plume mass observed downwind of the source. For a plume downwind of a point source, the source rate is determined by the plume mass above background and the residence time of methane in the plume. For a methane plume with uniform transport, residence time can be expressed as a simple ratio of the mean wind speed and the plume terminal distance. However, in a real-world scenario the plume dissipates downwind of the source due to turbulent diffusion and the mean wind speed is replaced by an effective wind speed (U_eff_) related to the observed wind speed and the spatial extent of the plume. The determination of plume extent depends on how effectively a plume can be separated from the background methane concentration on a pixel-by-pixel basis.

To separate simulated methane plumes from the background, we follow a two-step procedure based on Varon et al.^5^. First, we compared the mean methane plume within a 5 × 5 50 m pseudo pixel neighborhood centered on the pixel of interest with respect to its 5 × 5 pseudo pixel background methane concentration using the student’s t-test at the 95% (2-sigma) confidence level. The pixels satisfying the t-test are assigned a unit value and the remainder are assigned a zero value. The second step filters the pixels with random classification errors to create a Boolean plume mask. The resulting plume mask is smoothed with a 3 × 3 pseudo pixel median filter followed by convolution with a 2-dimensional Gaussian filter and subsequent thresholding^5^. The resulting plume mask is then used to calculate the surface area of the detectable plume. The plume extent is defined as the square root of the mask area.

We determine U_eff_ using two constant unit emission rate tracers (Tracers 23 and 24) emitted from two different pads. For these tracers the variability in their methane column is driven purely by turbulence making them suitable for determining U_eff_. The mean U_eff_, determined from Tracer 23, 24 was found to be ~ 2.6 m/s with mean 10 m wind speeds of ~ 4 m/s for both constant tracers with 1-s plumes. The U_eff_ for 10-min averaged plumes is ~ 3.2 m/s. In this study, the 10 m wind speeds used to derive U_eff_ are taken from WRF-LES model output. We acknowledge that such simulations may not always be available in the real-world scenarios. In such cases, local meteorological/reanalysis data may be used together with an error analysis. The U_eff_, IME, and the plume extent are then used to estimate time series of source rate (Q) for all sources at the seven well pads. The error propagation method (see supplementary material for details) is used to estimate source rate errors corresponding to different scenarios.

## Results and discussion

Figure 1a–c shows the model domain with three different pseudo-satellite pixels and the location of seven well pads, the corresponding spatial distribution of WRF-LES simulated surface methane mixing ratios at 01:20:00 UTC, and the time series of OFES emissions from seven well pads on Jan 01, 2017, at 01:00–03:00 UTC, respectively. Analysis of two-hour averaged WRF-LES model profiles at 3 km, 1 km, and 50 m spatial resolution for four scenarios described above shows that the major change in WRF-LES simulated methane plumes was mostly limited to ~ 50 m above ground in all the cases (Fig. S2). The maximum surface methane levels including all sources were about 3.2 ppm, 9.5 ppm, and 618.1 ppm, respectively for 3 km, 1 km, and 50 m pixels. The exclusion of liquids unloading events from Pad-1 and 4 reduced the maximum surface methane levels to 2.0 ppm, 2.1 ppm and 8.9 ppm, respectively, for 3 km, 1 km, and 50 m pixels. The mean background methane level was 1.98 ppm for all the pixels.

### Methane column enhancements

**Figure 2:**
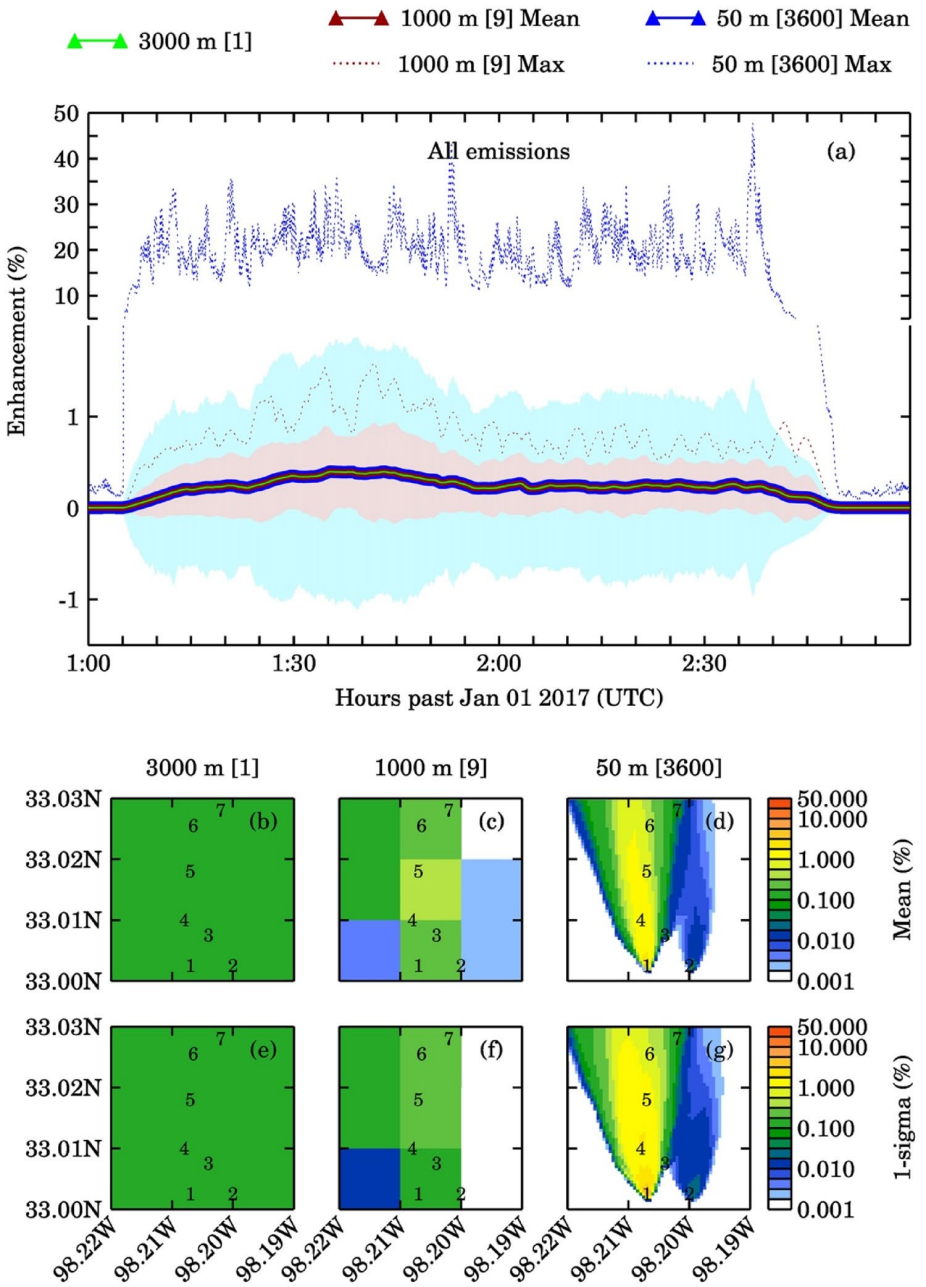
(**a**) Time series of percentage methane column enhancement with all emissions sources during the two-hour simulation period for three pseudo satellite pixels (there are 3600, 50 m pixels; 9, 1000 m pixels in one 3000 m pixel). Light green, dark red, and dark blue solid lines represent mean values and dotted lines represent maximum values over 3000 m, 1000 m and 50 m pixels, respectively. The plot also shows 1-sigma standard deviation as shaded area (light brown for 1000 m and light blue for 50 m pixels). Plots (**b**–**d**) represent the temporal averaged spatial distribution of column enhancements for three satellite pixels and plots (**e**–**g**) represent the 1-sigma variations to the mean column enhancements. The numbers mentioned in the square brackets are the number of pixels for each resolution.

**Figure 3:**
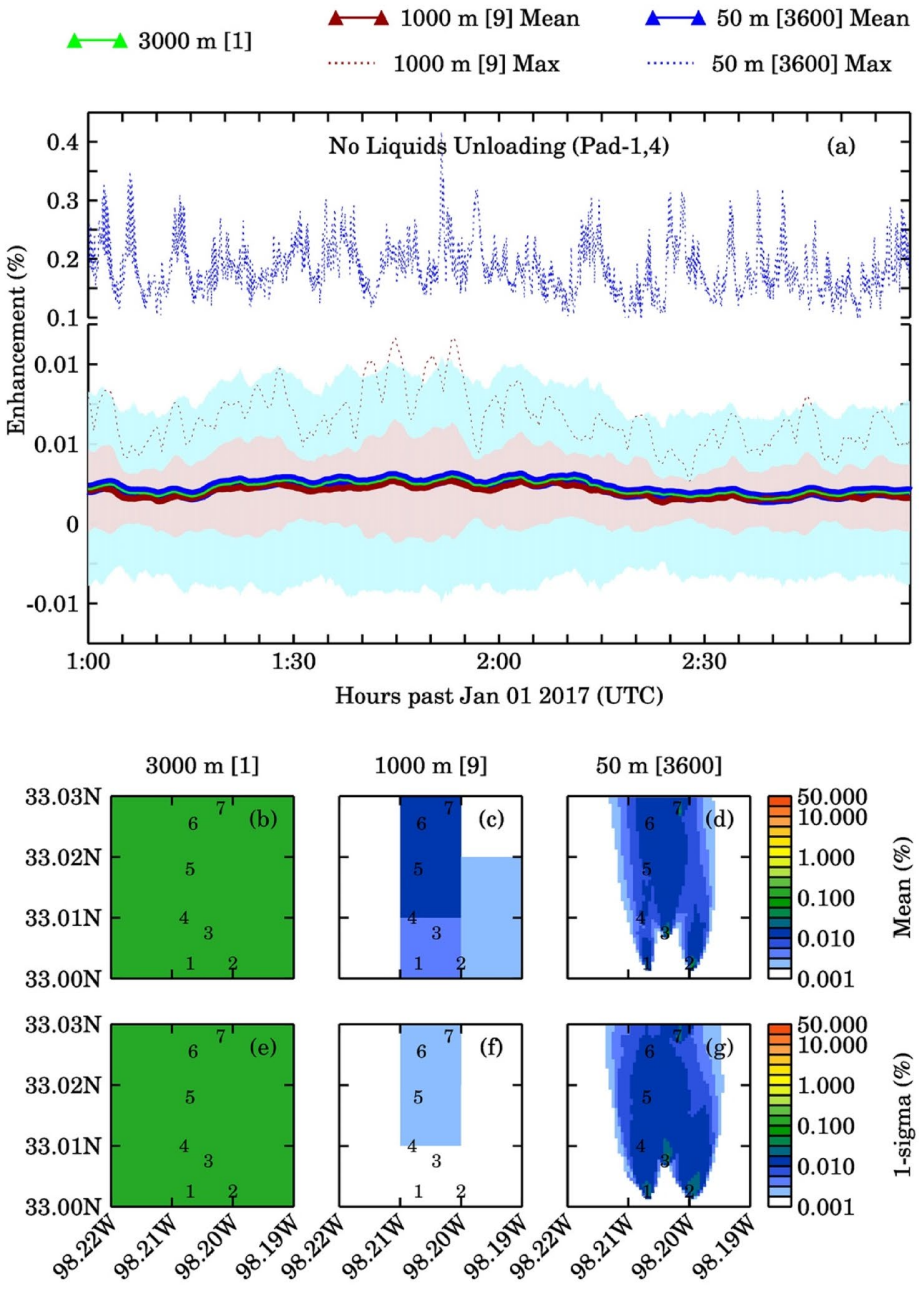
(**a**) Time series of percentage methane column enhancement with all emissions sources excluding the liquids unloading emissions from Pad-1 and 4 for three pseudo satellite pixels (there are 3600, 50 m pixels; 9, 1000 m pixels in one 3000 m pixel). Light green, dark red, and dark blue solid lines represent mean values and dotted lines represent maximum values over 3000 m, 1000 m and 50 m pixels, respectively. The plot also shows 1-sigma standard deviation as shaded area (light brown for 1000 m and light blue for 50 m pixels). Plots (**b**–**d**) represent the temporal averaged spatial distribution of column enhancements for three satellite pixels and plots (**e**–**g**) represent the 1-sigma variations to the mean column enhancements. The numbers mentioned in the square brackets are the number of pixels for that resolution.

The average methane column (with 1-sigma standard deviation) from all emission sources and the corresponding background were 3.79 ± 0.04 × 10^19^ molecules/cm^2^ and 3.78 × 10^19^ molecules/cm^2^, respectively, for the 50 m pseudo pixels. Larger pixels showed smaller standard deviations around the mean. The percent methane enhancement for ALL and NOPAD14 emission scenarios (noise free case) at three pseudo pixels are shown in Figs. 2 and 3, and the column enhancements corresponding to other two emissions scenarios viz., NOPAD1, NOPAD4 are shown in Figs. S3–S4. The maximum enhancement percentage increases by about fourfold and 120-fold as we increase the spatial resolution from 3 to 1 km and 3 km to 50 m, respectively. All pseudo pixel resolutions affected by the liquid unloading emissions (Figs. 2, S3, and S4) exhibit a large influence of turbulence induced variability on methane column. The 50 m pseudo pixels downwind of well pads 1 and 4 showed the maximum column enhancements, with changes of ~ 2 orders of magnitude. Another key observation is, in the absence of liquid unloading, the minimal methane concentration enhancements make it difficult to detect the sources above background columns with enhancements of 0.01%, 0.02%, and 0.42% for 3 km, 1 km, and 50 m pseudo pixels, respectively. In contrast, the maximum column enhancements above the background (for scenarios with liquids unloading emission) were about 0.4%, 1.6%, and 47.8%, respectively. Table S2 presents details of methane columns and their enhancements with respect to background for all the scenarios and resolutions.

### IME derived source rates

**Figure 4:**
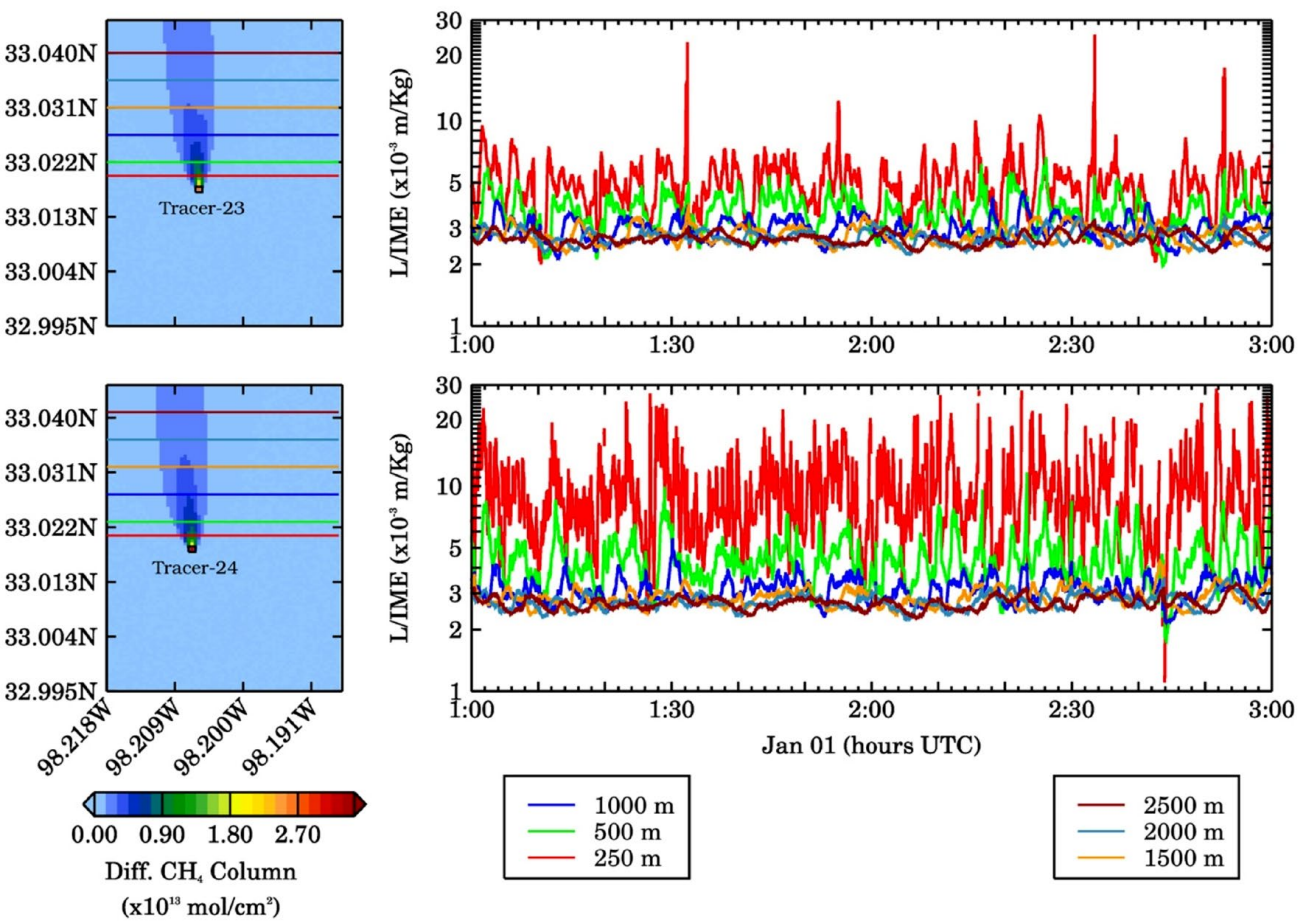
(Left) Spatial distribution of averaged methane column for constant emission sources (tracers 23, top; 24, bottom) over the model domain. The solid-colored lines represent 6 different intersections at 250, 500, 1000, 1500, 2000, and 2500 m downwind of the methane plume. (Right) Ratio of effective plume length to IME for Tracer-23 (top-right) and Tracer-24 (bottom-right), respectively.

The column enhancements for 50 m pseudo pixels were used to estimate emission source rates using the IME method for each well pad. The 50 m pseudo pixels were selected because the coarser resolution pseudo pixels did not produce a large enough methane enhancement to be visible above typical satellite noise levels. One concern in the application of the IME method was the loss of mass at the northern boundary due to the smaller spatial extent of the domain, which could potentially bias the source rate estimation. A sensitivity test to check the ratio of plume extent (L) to IME, i.e., L/IME was performed from the source to downwind distances of 250 m, 500 m, 1000 m, 1500 m, 2000 m, and 2500 m. At each of the six cutoff distances, the L/IME ratio for Tracers 23 and 24 were calculated and are shown in Fig. 4. For both the tracers, the L/IME ratio asymptotes as we moved further downwind, suggesting that the loss of mass at the northern boundary is not an issue for using the IME method. This also indicates that as we move further from the source of a plume, the mass contained within the plume becomes decoupled from the wind speed at the source.

**Figure 5:**
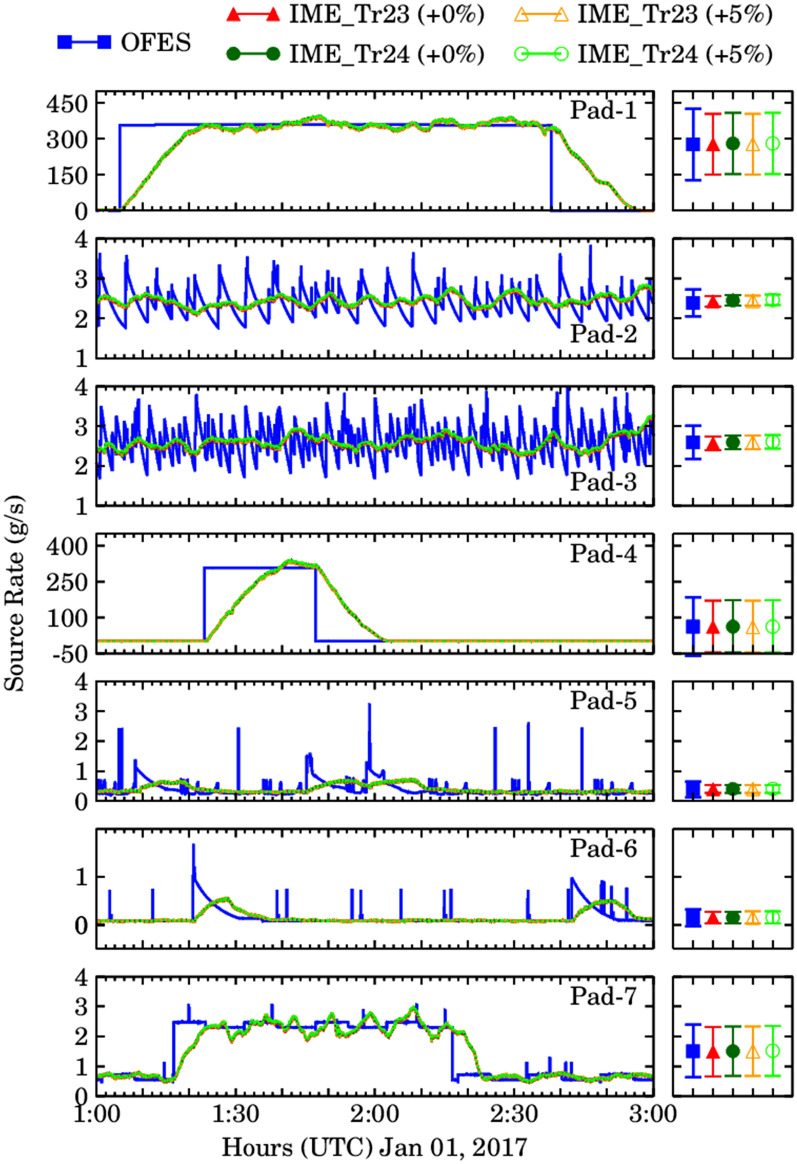
The comparison of OFES source rates (blue), IME derived source rates with no noise scenarios (red) and IME derived source rates with 5% random noise scenario (orange) over seven well pads from top to bottom using 1-s plumes. Different symbols represent source rates determined from different effective wind speed using tracer 23 (IME_TR23, shown as triangles) and tracer-24 (IME_TR24, shown as circles).

The plume extent, IME, and U_eff_ were used to estimate source rates for all seven well pads which are then compared with the known OFES emissions every second (Fig. 5) and 10-min average plumes (Fig. S5). The 10-min average was chosen because the bias in WRF-LES simulated wind speed at this time scale is less than 1 m/s and does not exceed 1 m/s for averaging time longer than 10 min. Further details of model wind speed calculations are provided in the supplementary material. The IME estimated emission rate for the noise free signal including all 22 sources over the two-hour simulation overestimated the simulated emissions by only 0.2–1.1% for 1 s plumes and 5.3–6.4% for 10-min average plumes. The emission rates corresponding to different noise (≤ 5%) levels differ by 0.3–0.5% for 1-s plumes and 6.9–10.2% for 10-min average plumes. Since the random noise is additive, the bias tends to increase with increase in noise levels from pure signal to + 5% random noise. Additional analysis by considering 25% errors in methane retrievals showed that the corresponding changes for single plumes were 10.0–11.6% for the seven well pads. The overestimation in IME derived source rates is also higher for 10-min average plumes, which suggests in well mixed plumes the rate of detection could be higher than instantaneous every second plumes. The mean IME derived source rates with errors for 1-s and 10-min averaged plumes with two different effective wind speed estimates are presented in Table 1. During the two-hour simulation period, the IME estimates showed a high degree of variability due to atmospheric turbulence. Overall, the mean IME derived source rates averaged over the two-hour simulation were like the mean OFES source rates, and the methodology worked very well to capture the average emissions aggregated over time, however, there are some limitations with this approach. The first limitation is the amount of time it typically required (~ 20 min for Pad-1, Pad-4; Figs. 5 and S5) to accurately estimate the magnitude of the large emission plumes corresponding to liquid unloading events. To detect and accurately estimate any such sources, time is required to build sufficient mass downwind of the source. Importantly, if a satellite scan is before this time, it will likely underestimate the source rate. A similar time lag is also observed when liquid unloading event end due to the time required for the enhanced mass to dissipate. Thus, the IME method is unable to detect rapid emission fluctuations common during normal operations, i.e., in the absence of liquid unloading emissions (for Pad-2, 3, 5, 6, and 7; Figs. 5, S5), this detection is further limited by the amount of noise associated with retrievals.

**Table 1:** Mean values with 1-sigma variations in OFES/IME derived source rates, respectively at seven well pads.

Pad #	OFES	IME (no noise) tracer-23/24	IME (± 1% noise) tracer-23/24	IME (± 5% noise) tracer-23/24
**1-s plumes**				
Pad-1	276.85 ± 149.25	277.40 ± 127.57/279.81 ± 128.67	278.57 ± 128.15/280.79 ± 129.17	278.08 ± 127.88/280.23 ± 128.88
Pad-2	2.39 ± 0.34	2.43 ± 0.17/2.45 ± 0.17	2.45 ± 0.18/2.47 ± 0.18	2.45 ± 0.18/2.46 ± 0.18
Pad-3	2.59 ± 0.41	2.57 ± 0.19/2.59 ± 0.19	2.59 ± 0.19/2.61 ± 0.19	2.59 ± 0.19/2.61 ± 0.19
Pad-4	61.41 ± 122.47	61.60 ± 108.57/62.28 ± 109.78	61.93 ± 109.15/62.54 ± 110.23	61.81 ± 108.94/62.43 ± 110.03
Pad-5	0.39 ± 0.26	0.40 ± 0.13/0.40 ± 0.13	0.40 ± 0.13/0.41 ± 0.13	0.40 ± 0.13/0.41 ± 0.13
Pad-6	0.14 ± 0.18	0.15 ± 0.13/0.15 ± 0.13	0.15 ± 0.13/0.15 ± 0.13	0.15 ± 0.13/0.15 ± 0.13
Pad-7	1.51 ± 0.88	1.49 ± 0.85/1.51 ± 0.86	1.51 ± 0.86/1.52 ± 0.87	1.51 ± 0.86/1.52 ± 0.87
**10-min average plumes**				
Pad-1	298.07 ± 121.59	316.84 ± 102.96/318.71 ± 103.56	337.56 ± 109.35/330.85 ± 107.18	346.23 ± 112.15/339.3 ± 109.91
Pad-2	2.38 ± 0.04	2.56 ± 0.13/2.55 ± 0.13	2.68 ± 0.2/2.63 ± 0.2	2.72 ± 0.2/2.67 ± 0.2
Pad-3	2.59 ± 0.05	2.64 ± 0.14/2.65 ± 0.14	2.74 ± 0.21/2.68 ± 0.21	2.8 ± 0.22/2.74 ± 0.21
Pad-4	66.95 ± 114.64	67.93 ± 108.52/69.97 ± 111.79	74.08 ± 117.48/72.57 ± 115.08	77.99 ± 123.68/76.35 ± 121.09
Pad-5	0.40 ± 0.13	0.41 ± 0.11/0.41 ± 0.11	0.43 ± 0.14/0.43 ± 0.14	0.45 ± 0.15/0.44 ± 0.14
Pad-6	0.15 ± 0.11	0.15 ± 0.11/0.15 ± 0.11	0.15 ± 0.12/0.15 ± 0.12	0.16 ± 0.12/0.15 ± 0.12
Pad-7	1.59 ± 0.82	1.46 ± 0.74/1.47 ± 0.75	1.48 ± 0.76/1.45 ± 0.74	1.52 ± 0.78/1.49 ± 0.76

IME derived source rates are provided for three noise scenarios, no noise, + 1% noise and + 5% noise. The two source rates are estimated using effective wind speed from Tracers-23/24 using 1-s (top) and 10-min average plumes (bottom).

## Conclusions

Our results suggests that two orders of magnitude change in emission rates at the surface results only in about 0.4%, 1.6%, and 47.8% enhancement in the methane column observed by the satellites at 3 km, 1 km, and 50 m, resolutions, respectively. The large, persistent point sources (e.g., liquid unloading events) could be detected using state of art instruments with sufficient resolution (e.g., with 50 m pixels shown here) using the IME methodology. However, our results also show that sources associated with normal operations, excluding liquid unloading events, that are rapidly fluctuating, are difficult to accurately quantify provided the limitations in satellite detection limits, precision, overpass timing, and pixel resolution. Our analysis also shows that detection would in principle be limited by the strength of emission source and atmospheric turbulence. With current LEO satellites, the time of scan with respect to persistent emissions and corresponding plume buildup is an important factor in detectability of any source. This time limitation could be removed by the continuous monitoring using geostationary satellites, though these types of satellites are limited by the loss in signal strength due to their large distance from Earth. Our results indicate a continued need for coordinated space-aircraft/ground measurements to fully characterize all emissions from O&G point sources (e.g., Zavala-Araiza et al.^16^), as well as importance of bottom-up/inventory estimates^17^ that could provide accurate emissions estimates from the well pads provided they are not misrepresented in terms of facility count and characterization of sources^16^, including the smaller sources that are often undetectable by satellite-based measurements.

## Supplementary Information

Supplementary Information.

## Data Availability

The datasets generated and/or analyzed during the current study are not publicly available due their large size but are available from the corresponding author on reasonable request upon approval of the permission of the funding agency.
