# Supplementary material for: Evaluating the detectability of methane point sources from satellite observing systems using microscale modeling
Source: Sci Rep. 2022 Oct 19;12:17425. doi: 10.1038/s41598-022-20567-z (PMC9581893; doi:10.1038/s41598-022-20567-z)
Supplement: Supplementary file 1 — Supplementary Information. [file 41598_2022_20567_MOESM1_ESM.docx]

**Supplementary material to**

**Evaluating the detectability of methane point sources from satellite observing systems using microscale modeling**

Piyush Bhardwaj^1*^, Rajesh Kumar^1^, Douglas A. Mitchell^2^, Cynthia A. Randles^3^, Nicole Downey^4^, Doug Blewitt^4^, Branko Kosovic^1^

^1^ National Center for Atmospheric Research, Boulder, CO, USA

^2^ ExxonMobil Upstream Research Company, USA

^3^ ExxonMobil Research and Engineering Company, Annandale, NJ, USA

^4^ Earth System Sciences, LLC, Albuquerque, NM, USA

*Corresponding author: **piyushbhar@gmail.com**

**This supplement contains one text section, five figures and two tables.**

**Section S1: Calculation of methane columns, their enhancements, and source rate estimation (IME)**

To calculate total column methane, WRF-LES simulated methane profiles were extended to the top of the atmosphere using the Whole Atmosphere Community Climate Model (WACCM) climatology for Jan 2018. The complete merged methane profile over each pixel “i” (different pseudo satellite footprints) is used to estimate methane columns for a total of 25 different tracers identified as “k”, twenty-two of which (k = 1-22) represent different emission sources from seven well pads, two (k-23, 24) are constant emission tracers used for U_eff_ calculation, and one (k=25) is a background tracer. The methane column over pixel “i” and for tracer “k” is calculated by integrating methane mixing ratio ($x_{\mathrm{ij}}^{k}$) over the vertical grid using equation (1)

$\mathrm{TC}_{i}^{k}$= $\sum_{j=surf}^{Model Top} \frac{x_{\mathrm{ij}}^{k} N_{A} \Delta P}{M_{a} g}$ [molecules/cm^2^] (1)

where, $x_{\mathrm{ij}}^{k}$is the methane mixing ratio (in ppmv) for tracer “k” at pixel “i” between pressure $P_{j}$and$P_{j+1}$, $N_{A}$ is Avogadro’s constant ($N_{A}$= 6.022 x 10^23^ molecules/mol), $\Delta P$is pressure difference (i.e. $P_{j}$-$P_{j+1}$, g/cm-sec^2^) $M_{a}$ is molar mass of dry air ($M_{a}$= 28.96 g/mol) and g is gravitational acceleration (g = 981 cm/s). These columns are then used to calculate the difference columns over seven well pads for four different scenarios. We also estimated the percentage enhancement with respect to the background column in four different scenarios at three different pseudo satellite footprints.

$Enhancement= \frac{(\mathrm{TC}_{i}^{n} -\mathrm{TC}_{i}^{\mathrm{bkg}})}{\mathrm{TC}_{i}^{\mathrm{bkg}}}$ [%] (2)

The four different scenarios “n” are (1) sum of all “k” tracers [k = 1-22] representing methane column enhancement due to all emission sources within our domain (ALL) (2) sum of all tracers excluding tracers corresponding to liquid unloading emissions from pad-1 and pad-4 [k = 3, 17] (NOPAD14), (3) sum of all tracers excluding tracers corresponding to liquid unloading emissions from pad-1 [k = 3] (NOPAD1) and (4) sum of all tracers excluding tracers corresponding to liquid unloading emissions from pad4 [k = 17] (NOPAD4). Methane columns are calculated at native 10 m resolution, which are then averaged to create pseudo satellite footprints at the spatial resolution of 3 km x 3 km, 1 km x 1 km and 50 m x 50 m. The detailed description of the 25 tracers over seven pads and four scenarios (combination of tracers) is presented in Table-S1.

**Integrated mass enhancement and source rate:** The behavior of methane plumes from oil and gas production wells are characterized by atmospheric turbulence, local winds and strength of the emission source. For this study we only used the results from the highest resolution pseudo satellite footprints (50 m x 50 m). The source rate in the integrated mass enhancement ~~IME~~ method is related to the total downwind column mass of the plume. The details provided here to estimate source rate based on integrated mass enhancement method described in Varon et al^1^. For a plume downwind of a point source having m pixels of area A_i_ (i = 1,.., m), the source rate is calculated as,

$Q =\frac{\mathrm{IME}}{\tau}$ [Kg/sec] (3)

where τ is the residence time of methane in the plume. For a methane plume with uniform transport, τ could be expressed as a ratio of its mean wind speed and terminal distance. However, for a real-world scenario the plume dissipation downwind of an oil pad is due to turbulent diffusion and hence, τ =$\frac{L}{\mathrm{Ueff}}$ where, U_eff_ is a variable related to the observed wind speed and L is the extent of the plume. The determination of plume extent is dependent on how effectively plume is separated from the background on a pixel level.

**Plume mask, plume area and its extent:** To separate synthetic methane plumes from background, we defined a plume mask using a two-step procedure following Varon et al^1^. First, we compared the mean methane plume at 5 x 5 (50 m) pseudo pixel neighborhood surrounding each pixel “i” with respect to its 5 x 5 pseudo pixel background using the student’s t-test at 95% (2-sigma) confidence level. The pixels which satisfy (p < 0.05) the t-test are assigned a unit value and rest pixels are assigned a zero value. The second step is to filter the pixels with random classification errors to create a Boolean plume mask. For filtering, we smooth the plume mask with a median filter followed by a gaussian filter and thresholding. The median filter is created over 3 x 3 pseudo pixel neighborhoods surrounding each pixel and it is then convolved with a 2-dimensional gaussian filter with 2-sigma standard deviation. The resulting plume mask is then used to calculate the number of pixels where a detectable methane plume is present. Each pixel in this study has a constant area (A_i_ = 2500 sq. m) and total area of the plume $A_{m}$ is the number of plume pixels times the pixel area A_i_. The extent of plume (L) is defined as the square root of the plume area corresponding to the plume downwind of each pad/scenario.

$L = \sqrt{A_{m}}$ [m] (4)

Once the plume area and number of plume pixels are known, IME is estimated (equation 5) by accumulating column mass enhancement corresponding to each pixel with area A_i_,

IME = $\sum_{i=1}^{m} \Delta\Omega_{i}* A_{i}$ [Kg] (5)

where, column mass enhancement ($\Delta\Omega_{i}$) at each pixel “i” is estimated using the difference columns (discussed in previous section) over each pixel “i” and tracer “k”,

$\Delta\Omega_{i}$= ($\mathrm{TC}_{i}^{n}$ - $\mathrm{TC}_{i}^{\mathrm{BKG}}$) * $\frac{M_{CH4}}{N_{A}}$ [Kg/m^2^] (6)

where “n” is the different combination of tracers (k) that represent different pads, scenarios or individual tracers, respectively, discussed earlier and $M_{CH4}$is the molar mass of CH_4_ ($M_{CH4}$= 16.04 x 10^-3^ Kg/mol).

**WRF-LES 10 m wind speed validation and effect of time integration of plumes:** The determination of U_eff_ is performed using WRF-LES derived 10 m wind speeds following Varon et al^1^. In this study the validation of WRF-LES 10 m wind speed is not performed explicitly, however, a similar WRF-LES setup with daytime convective boundary layer with imposed geotropic winds (U ~ 6.5 m/s) was shown to capture both wind speed and its variability at 10 m above the ground level very well^2^. The study compared WRF simulation to SWIFT tall (200 m) tower mounted sonic anemometer measurements at Texas Tech University and showed that the sonic anemometer error is about 0.1 m/s, the LES error should not be much larger than that. In another study performed by our group^3^, an excellent agreement is observed between WRF simulations and observations (at 40 m AGL), in mesoscale to microscale simulations of flow over flat terrain, both in terms of time averaged wind speed and fluctuations and if the large-scale forcing is captured well, then under convective conditions the 10 m wind speed is accurately predicted. The bias in wind speed decreases as the averaging time is increased and at an averaging time of ~10 mins the biases in 10 m wind speed is <1 m/s. Considering above, the impact of integration time on source rate is assessed with 10 mins (600s) running averaged plumes. The results are presented in table 2 and Fig. S5.

**Error analysis:** Using the Source rate and U_eff_ relation, the error propagation method is used to estimate the errors at 1 sec and 10 min integration time (600 sec). The source rate using IME method is determined as,

$Q =\frac{IME*Ueff}{L}$ [Kg/sec] (7)

Using error propagation, the error in Q (δQ) would be,

$\delta Q=Q_{\mathrm{mean}}* \sqrt{\left( \frac{\delta IME}{\mathrm{IME}_{\mathrm{mean}}} \right)^{2}+\left( \frac{\delta L}{L_{\mathrm{mean}}} \right)^{2}+\left( \frac{\delta Ueff}{\mathrm{Ueff}_{\mathrm{mean}}} \right)^{2}}$ [Kg/sec] (8)

where, $\delta(IME)$, and $\delta\left( L \right)$are 1-sigma variations in IME and L during the simulation period for a given averaging time. $\mathrm{IME}_{\mathrm{mean}}, L_{\mathrm{mean}},$and $\mathrm{Ueff}_{\mathrm{mean}}$are the mean values during two-hour simulation time. The U_eff_ is related to 10 m wind speed using an empirical relation for two constant tracers at different averaging time and noise levels.

$Ueff=a log\left( ws10 \right)+b$ [m/s] (9)

Using error propagation, the error in U_eff_ ($\delta U\mathrm{eff}$) will be,

$\delta Ueff \cong|\frac{a* \delta ws10}{\mathrm{ws}{10}_{\mathrm{mean}}*ln(10)}|$ [m/s] (10)

Where, $\delta ws10$ is 1-sigma variation in $ws10$. The two extremum noise levels (1%-5%) are used to estimate Ueff, and $\delta Ueff from \delta ws10 and ws{10}_{\mathrm{mean}}.$ Since two tracers are used to estimate U_eff_, both source rate estimations values with errors are presented in the Table-S2 at 1-sec and 10-min averaging time. Apart from the errors above, the methane column fields determined from our method could also include additional errors (8-25%) in measurement columns including errors due to aerosol extinction^4^. The WRF-LES derived 10 m winds as used in this study may not be available in a real-world scenario. In such cases, the local meteorological/reanalysis data may be used, which can introduce larger uncertainty^1^ in the source rate determination.

**References:**

1. Varon, D. J. et al. Quantifying methane point sources from fine-scale satellite observations of atmospheric methane plumes. Atmospheric Measurement Techniques **11**, 5673–5686 (2018).
2. Mirocha, J. D., et al., Large-eddy simulation sensitivities to variations of configuration and forcing parameters in canonical boundary-layer flows for wind energy applications, Wind Energ. Sci., 3, 589–613 (2018).
3. Munoz-Esparza, D., et al. coupled mesoscale-LES modeling of a diurnal cycle during the CWEX-13 field campaign: From weather to boundary-layer eddies, J. Adv. Model. Earth Syst., 9, 1572–1594 (2017).
4. Jervis, D., et al. The GHGSat-D imaging spectrometer, Atmos. Meas. Tech., 14, 2127–2140 (2021).

**Availability of Data and Materials:** The datasets generated and/or analyzed during the current study are not publicly available due their large size but are available from the corresponding author on reasonable request upon approval of the permission of the funding agency.

**
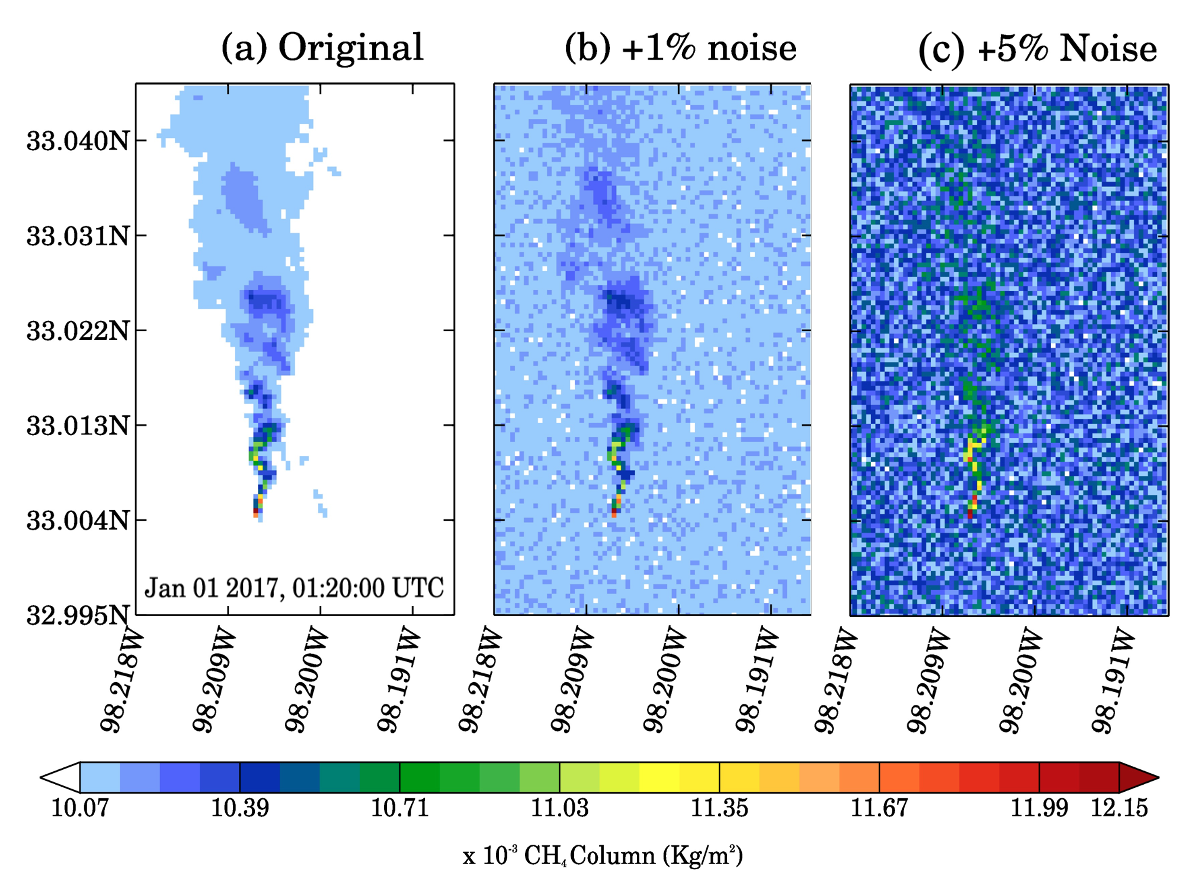
**

**Figure S1:** Spatial distribution of methane column with (a) no noise, (b) 1 % noise, and (c) 5 % noise at 01:20:00 hours UTC including all tracers (SAT).

**
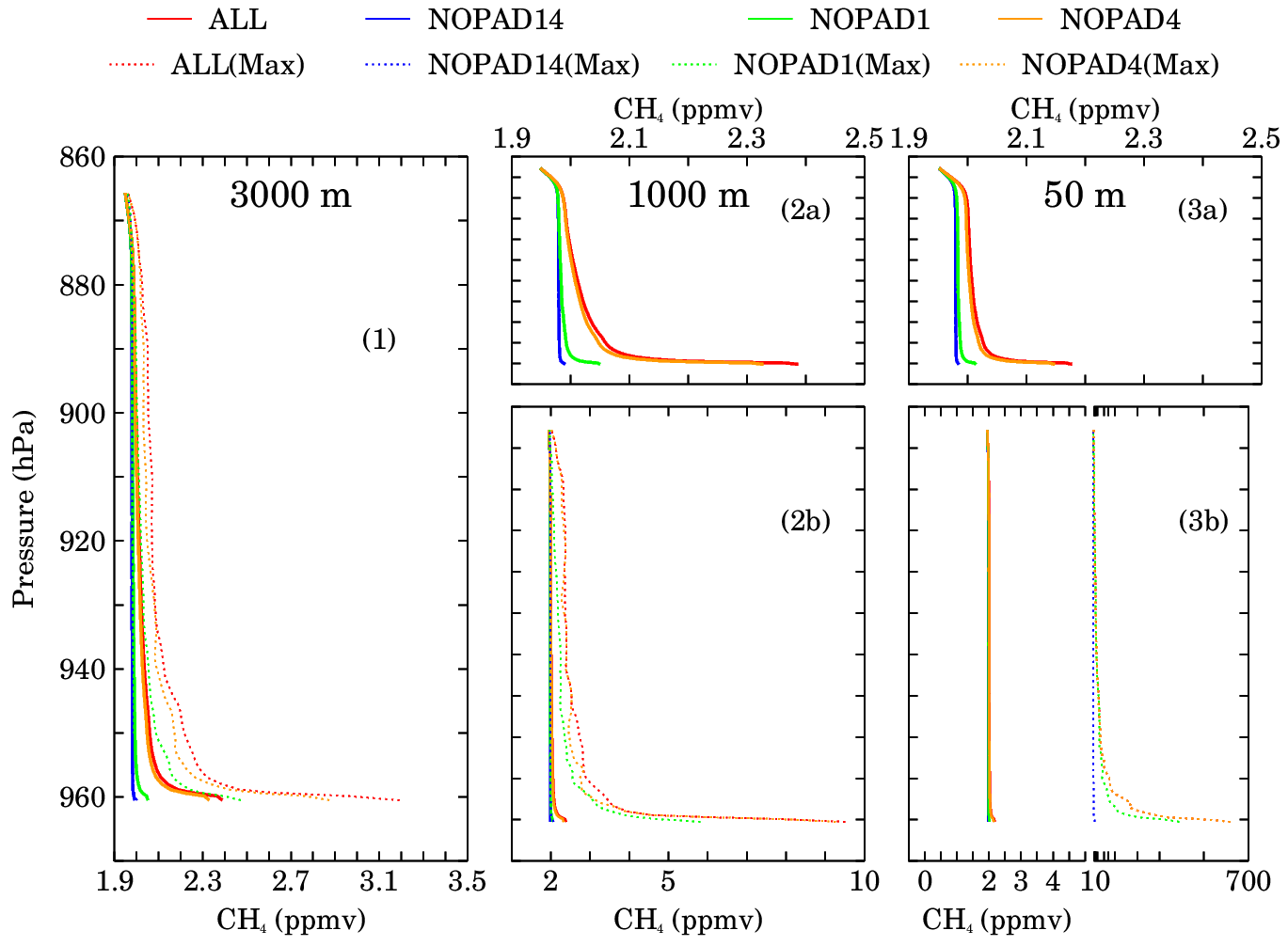
**

**Figure S2:** The vertical distribution of methane over 3000 m (1), 1000 m (2), and 50 m (3) pseudo satellite footprint in four different scenarios. The top right (a) panel show average methane profiles and bottom right (b) panels show methane profiles with averaged and maximum values, respectively.

**
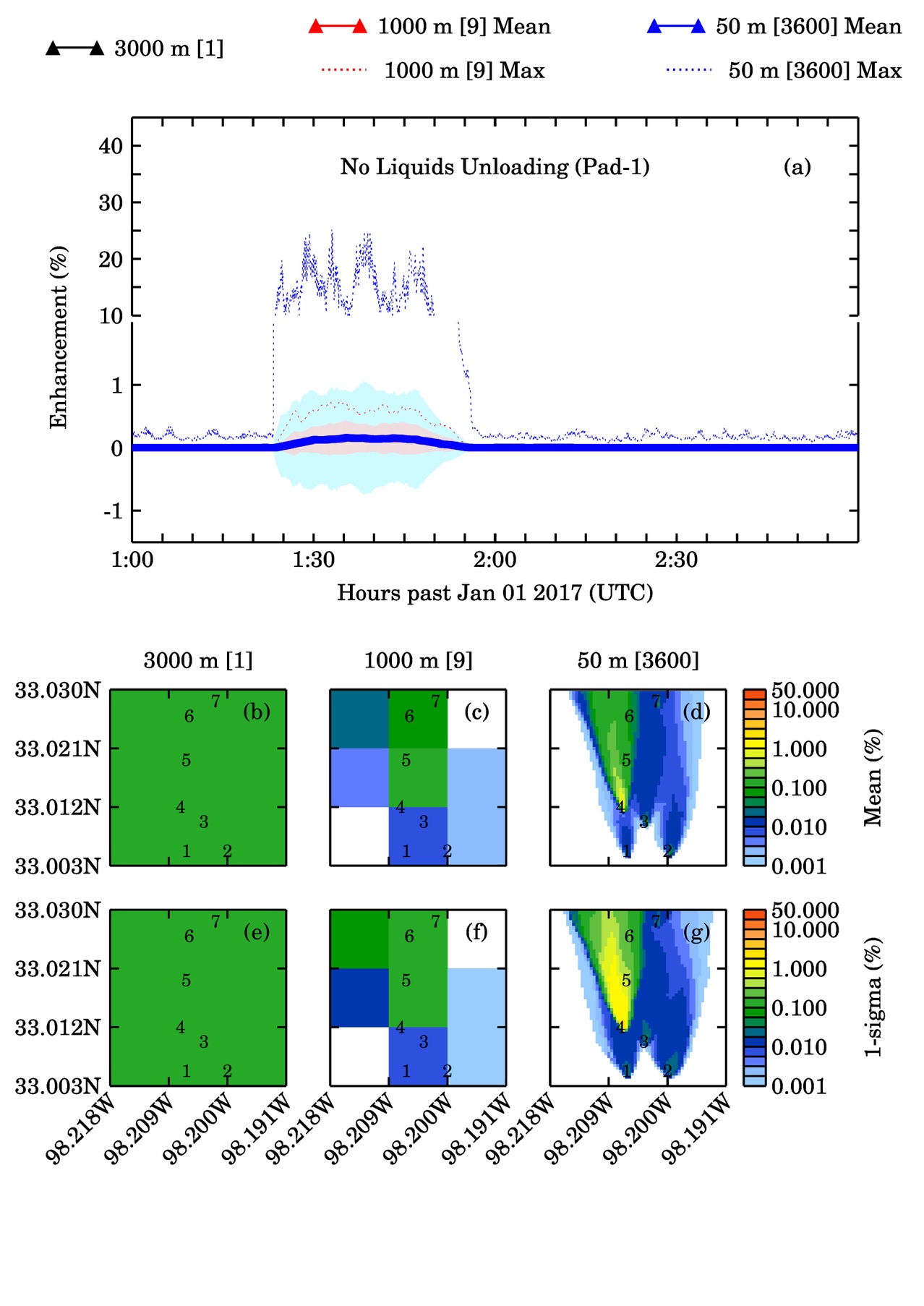
**

**Figure S3:** Top to bottom, (a) time series of percentage methane column enhancement with all emissions sources excluding the liquids unloading emissions from Pad-1 during the two-hour simulation period for three pseudo satellite pixels (solid lines). The plot also shows 1-sigma standard deviation (shaded area) and maximum values (dotted lines). Plots (b-d) represent the temporal averaged spatial distribution of column enhancements for three satellite pixels and plots (e-g) represent the 1-sigma variations to the mean column enhancements.

**
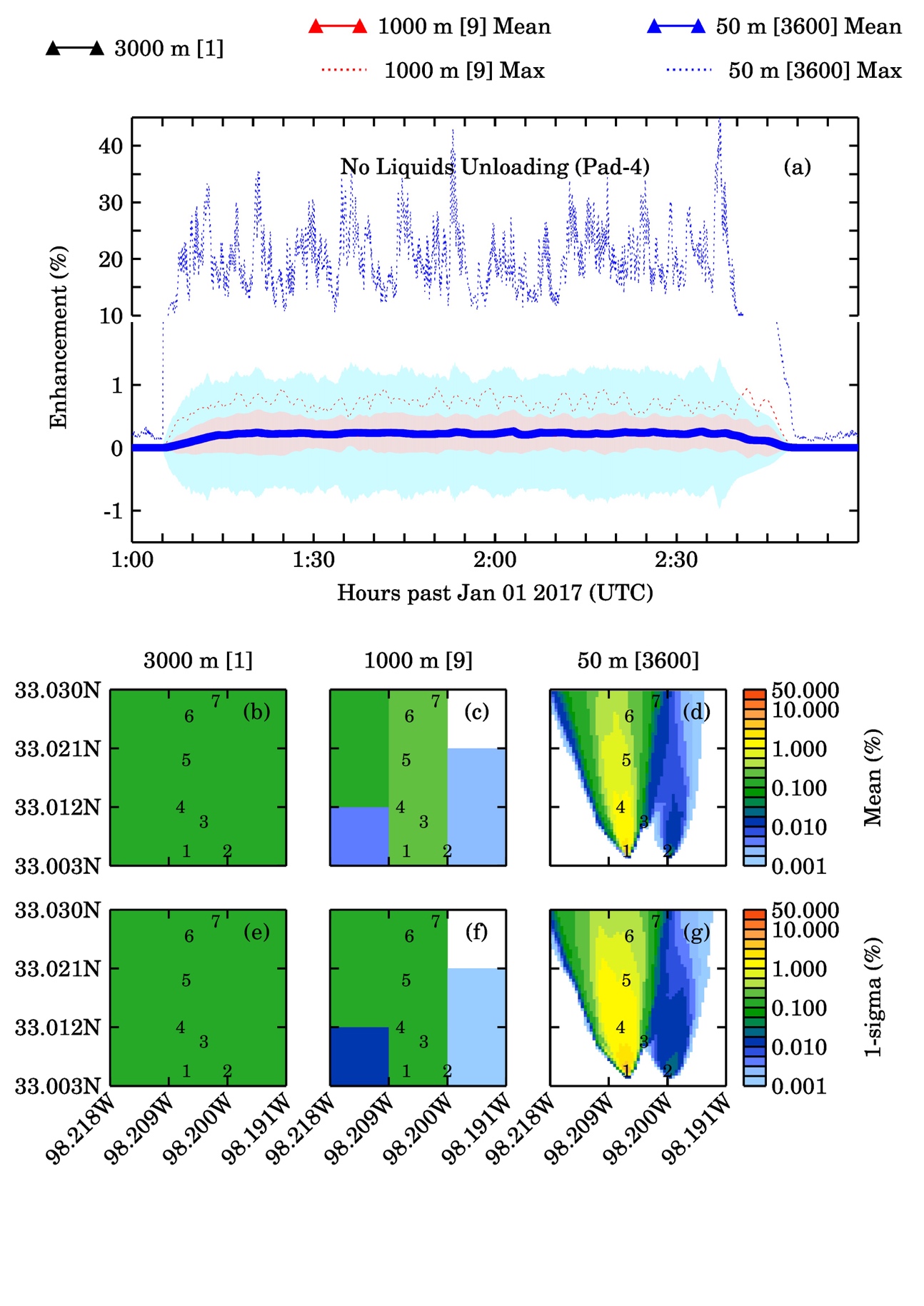
**

**Figure S4:** Top to bottom, (a) time series of percentage methane column enhancement with all emissions sources excluding the liquids unloading emissions from Pad-4 during the two-hour simulation period for three pseudo satellite pixels (solid lines). The plot also shows 1-sigma standard deviation (shaded area) and maximum values (dotted lines). Plots (b-d) represent the temporal averaged spatial distribution of column enhancements for three satellite pixels and plots (e-g) represent the 1-sigma variations to the mean column enhancements.

**
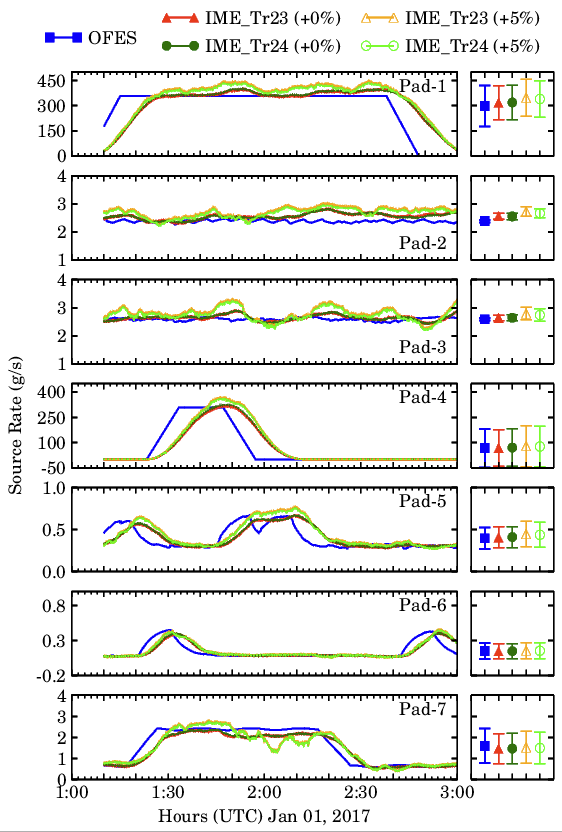
**

**Figure S5:** The comparison of OFES source rates (blue), IME derived source rates with no noise scenarios (red) and IME derived source rates with 5% random noise scenario (orange) over seven well pads from top to bottom using 10-min average plumes. Different symbols represent source rates determined from different effective wind speed using tracer 23 (IME_TR23, shown as triangles) and tracer-24 (IME_TR24, shown as circles).**Table S1:** Description of different combinations of WRF-LES tracers (refer Table-1) representing emissions/columns in four scenarios.

| **Pad # / Scenarios** | **Tracer #** | **Tagged emission source** |
| --- | --- | --- |
| Pad - 1 | Tr 1 | Tank flashing |
|  | Tr 2 | CI Pump, Pneumatics |
|  | Tr 3 | Liquids Unloading |
|  | Tr 4 | Compressor |
|  | Tr 5 | Tank flashing |
| Pad - 2 | Tr 6 | Tank flashing |
|  | Tr 7 | Dehydrator |
|  | Tr 8 | CI Pump, Pneumatics |
|  | Tr 9 | Tank flashing |
| Pad - 3 | Tr 10 | Tank flashing |
|  | Tr 11 | CI Pump, Pneumatics |
|  | Tr 12 | Tank flashing |
| Pad - 4 | Tr 13 | Tank flashing |
|  | Tr 14 | CI Pump, Pneumatics |
|  | Tr 15 | Compressor |
|  | Tr 16 | Tank flashing |
|  | Tr 17 | Liquids Unloading |
| Pad - 5 | Tr 18 | Tank flashing |
|  | Tr 19 | CI Pump, Pneumatics |
| Pad - 6 | Tr 20 | Tank flashing |
|  | Tr 21 | CI Pump, Pneumatics, Compressor |
| Pad - 7 | Tr 22 | CI Pump, Pneumatics, Compressor |
| -- | Tr-23 | Constant emission |
| -- | Tr-24 | Constant emission |
| -- | Tr 25 | Background methane |
| ALL | [(Tr 1:22) + Tr 25] | All emissions sources including background |
| NOPAD14 | [(Tr 1:22) + Tr 25] - Tr (3,17) | All emissions excluding liquids unloading from Tracer # 3, 17 |
| NOPAD1 | [(Tr 1:22) + Tr 25] - Tr (3) | All emissions excluding liquids unloading from Tracer # 3 |
| NOPAD4 | [(Tr 1:22) + Tr 25] - Tr (17) | All emissions excluding liquids unloading from Tracer # 17 |

**Table S2:** The absolute methane column (x 10^19^ mol/cm^2^) and corresponding enhancement (%) for four different scenarios and background (BKG) for 3 different footprints over Barnett Shale in Texas. The top two values in each cell represent mean values with 1 sigma standard deviation and bottom two shown minimum and maximum values during the two-hour simulation period.

| **FP** | **Avg** ± **STD**  **Min Max** | **ALL** | **NOPAD14** | **NOPAD1** | **NOPAD4** |
| --- | --- | --- | --- | --- | --- |
| 3 km | Column  (x 10^19^ mol/cm^2^) | 3.79 ± 0.00  3.78 3.79 | 3.78 ± 0.00  3.78 3.78 | 3.78 ± 0.00  3.78 3.79 | 3.79 ± 0.00  3.78 3.79 |
|  | Enhancement (%) | 0.21 ± 0.12  0.00 0.40 | 0.00 ± 0.00  0.00 0.01 | 0.03 ± 0.05  0.00 0.16 | 0.18 ± 0.09  0.00 0.27 |
| 1 km | Column  (x 10^19^ mol/cm^2^) | 3.79 ± 0.01  3.78 3.84 | 3.78 ± 0.00  3.78 3.78 | 3.78 ± 0.00  3.78 3.81 | 3.79 ± 0.01  3.78 3.82 |
|  | Enhancement (%) | 0.21 ± 0.31  0.00 1.59 | 0.00 ± 0.00  0.00 0.02 | 0.03 ± 0.11  0.00 0.74 | 0.18 ± 0.26  0.00 0.97 |
| 50 m | Column  (x 10^19^ mol/cm^2^) | 3.79 ± 0.04  3.78 5.59 | 3.78 ± 0.00  3.78 3.80 | 3.78 ± 0.01  3.78 4.73 | 3.79 ± 0.03  3.78 5.59 |
|  | Enhancement (%) | 0.42 ± 1.30  0.00 47.82 | 0.01 ± 0.02  0.00 0.42 | 0.07 ± 0.49  0.00 25.19 | 0.36 ± 1.15  0.00 47.82 |
